# Biomass composition explains fruit relative growth rate and discriminates climacteric from non-climacteric species

**DOI:** 10.1093/jxb/eraa302

**Published:** 2020-06-27

**Authors:** Léa Roch, Sylvain Prigent, Holger Klose, Coffi-Belmys Cakpo, Bertrand Beauvoit, Catherine Deborde, Laetitia Fouillen, Pierre van Delft, Daniel Jacob, Björn Usadel, Zhanwu Dai, Michel Génard, Gilles Vercambre, Sophie Colombié, Annick Moing, Yves Gibon

**Affiliations:** 1 UMR 1332 Biologie du Fruit et Pathologie, INRAE, Univ. Bordeaux, INRAE Nouvelle Aquitaine – Bordeaux, Avenue Edouard Bourlaux, Villenave d’Ornon, France; 2 Institute for Biology, BioSC, RWTH Aachen University, Worringer Weg, Aachen, Germany; 3 Institute of Bio- and Geosciences, Plant Sciences (IBG-2), Forschungszentrum Jülich GmbH, Jülich, Germany; 4 UR 1115 PSH, INRAE, Avignon Cedex 9, France; 5 Bordeaux Metabolome, MetaboHUB, INRAE, Univ. Bordeaux, Avenue Edouard Bourlaux, Villenave d’Ornon, France; 6 UMR 5200, CNRS, Univ. Bordeaux, Laboratoire de Biogenèse Membranaire, Avenue Edouard Bourlaux, Villenave d’Ornon, France; 7 UMR 1287 EGFV, INRAE, Univ. Bordeaux, Bordeaux Sci Agro, Villenave d’Ornon, France; 8 CONICET - National University of La Plata, Argentina

**Keywords:** Biomass composition, climacteric, fruit, metabolism, metaphenomics, modelling, relative growth rate

## Abstract

Fleshy fruits are very varied, whether in terms of their composition, physiology, or rate and duration of growth. To understand the mechanisms that link metabolism to phenotypes, which would help the targeting of breeding strategies, we compared eight fleshy fruit species during development and ripening. Three herbaceous (eggplant, pepper, and cucumber), three tree (apple, peach, and clementine) and two vine (kiwifruit and grape) species were selected for their diversity. Fruit fresh weight and biomass composition, including the major soluble and insoluble components, were determined throughout fruit development and ripening. Best-fitting models of fruit weight were used to estimate relative growth rate (RGR), which was significantly correlated with several biomass components, especially protein content (*R*=84), stearate (*R*=0.72), palmitate (*R*=0.72), and lignocerate (*R*=0.68). The strong link between biomass composition and RGR was further evidenced by generalized linear models that predicted RGR with *R*-values exceeding 0.9. Comparison of the fruit also showed that climacteric fruit (apple, peach, kiwifruit) contained more non-cellulosic cell-wall glucose and fucose, and more starch, than non-climacteric fruit. The rate of starch net accumulation was also higher in climacteric fruit. These results suggest that the way biomass is constructed has a major influence on performance, especially growth rate.

## Teaser

A comparison of eight fruit species (herbaceous, vines, and trees) throughout their development shows that biomass composition and fruit relative growth rate are closely linked.

## Introduction

Understanding the mechanisms that link metabolism to crop phenotypes helps in targeting breeding strategies (Giovannoni, 2006). In fruit, resistance to biotic or abiotic stress during growth, development, and ripening, as well as flavour, nutritional value, and health benefits, are all affected by biomass composition. A key objective is therefore to understand the factors that influence the construction of biomass and to identify the trade-offs between quality and biomass production, in order to be able to propose targets for improving fruit species and crop management (Beauvoit *et al.*, 2018).

There are many types of fleshy fruit species: annual or perennial, and herbaceous, vines, shrubs, or trees. The dry biomass of fleshy fruit is mainly composed of cell-wall and non-structural carbohydrates. However, depending on the species and/or stage of development, fruit may contain significant amounts of organic acids, amino acids, proteins, and lipids (Coombe, 1976; Colombié *et al.*, 2015). Fleshy fruits are also an important source of specialized metabolites, including phenolic antioxidants (Rice-Evans *et al.*, 1997) and other antioxidants, such as ascorbate, which can reach high concentrations (Fenech *et al.*, 2019). The biochemical composition of fleshy fruits can vary greatly from one species to another (Coombe, 1976; Dai *et al.*, 2016) but also during fruit development (Carrari *et al.*, 2006; Dai *et al.*, 2013). To date, the link between fruit biomass composition and its growth rate and duration of development has been poorly understood. Apart from their importance in terms of taste, sugars and organic acids are widely considered essential for fruit cell expansion (e.g. Menu *et al.*, 2004; Beauvoit *et al.*, 2014). However, very sweet fruits such as strawberry or melon can develop in a few weeks while others, such as kiwifruit or clementine, need more than 200 days (Coombe, 1976).

Several publications have linked metabolic composition to plant performance, for example, rosette biomass in Arabidopsis (Meyer *et al.*, 2007), or several agronomic traits, including grain yield in maize (Riedelsheimer *et al.*, 2012), but links between species have been reported only rarely (Niemann *et al*., 1992, 1995). Most molecular and system-oriented research on fruit focuses on a particular species, such as tomato, which has become the model species for fleshy fruit (Tohge and Fernie, 2015). However, a comparison of primary metabolomes between four fruit species indicated that the best separation based on these profiles was between climacteric and non-climacteric fruits rather than between botanical families (Klie *et al.*, 2014). This indicates that the climacteric character, that is, a stage characterized by a peak in ethylene production and a respiration burst, which appeared independently within various families or even species (Paul *et al.*, 2012; Batista-Silva *et al.*, 2018), may have a greater effect on the metabolism than genetic distance.

In this study, we investigated whether fruit biomass composition is linked to traits of interest such as fruit relative growth rate (RGR). Highly diverse fruits from eight herbaceous, vine, or tree species, comprising both climacteric and non-climacteric fruits, were compared. The composition of the biomass was analysed using targeted and non-targeted analytical approaches, then the biomass components shared between species were selected and the stages of development standardized. Multilinear modelling was performed to search for possible links between biomass composition and fruit growth rate. Multivariate discriminant analyses were then used to associate metabolic variables with groups of species (herbaceous, vines, or trees; climacteric or non-climacteric).

## Materials and methods

### Fruit material

The study was carried out with pepper (*Capsicum annuum* L. cv Gonto Clause), eggplant (*Solanum melongena* L. cv Monarca RZ), peach (*Prunus persica* L. cv Nectarlove), apple (*Malus × domestica* Borkh. cv Golden), cucumber (*Cucumis sativus* L. cv Aljona), kiwifruit (*Actinidia deliciosa* Chev. cv Hayward), grape (*Vitis vinifera* L. cv Cabernet Sauvignon), and clementine (*Citrus clementina* hort. cv SRA 63). One variety was chosen for each species, based either on its commercial value or because the model has been studied for several years. Eggplant and pepper (Sainte Livrade, F), cucumber (Carquefou, F), and grapevine (Villenave d’Ornon, F) were grown in greenhouses, and kiwifruit (Sainte-Livrade, F), apple (Saint-Marcel-lès-Valence, F), peach (Avignon, F), and clementine (San Giuliano, F) were grown in orchards. All species were grown following commercial practices except grape, which was cultivated as fruiting cuttings (Ollat *et al.*, 1998).

Fruits at nine stages of development were collected from anthesis or very early stages after flowering (apple, peach, grape, and clementine) until physiological maturity, except for cucumber, which was sampled until commercial maturity. For each stage, identified with the corresponding number of days after anthesis (DAA), five biological replicates (except for the early stages of peach) were collected, with a minimum of four fruits per replicate (except for cucumber). This provided a total of 455 samples. Each fruit was immediately measured and weighed, then cut into small pieces and shock-frozen in liquid nitrogen. Samples were stored at –80 °C before cryogrinding (Spex Genogrinder 2010, Fisher Scientific, Illkirch, France), lyophilization (Dura Dry MP Freeze Dryer, Warminster, PA USA), and biochemical analyses. Lyophilization allowed the dry matter content to be measured. For each species, an additional sample, referred to as a ‘stage-mixture sample’ and used to identify major soluble compounds, was prepared by mixing equal amounts of all samples taken during fruit development.

### Biochemical analysis

Nuclear magnetic resonance spectroscopy (NMR) of the stage-mixture samples was used to identify the major metabolites in each fruit. Metabolites were extracted from 20 or 50 mg lyophilized powder with an ethanol–water series (Deborde *et al.*, 2009). Each pH-adjusted lyophilized extract containing 2 mM EDTA was solubilized and ^1^H-NMR spectra were recorded at 500.162 MHz (Bruker Avance III, Wissembourg, France). Annotation of the spectra was performed using published data (Capitani *et al*., 2010, 2013; Ritota *et al.*, 2010; Mulas *et al.*, 2011; de Oliveira *et al.*, 2014; Tomita *et al.*, 2015; Zhao *et al*, 2017), databases [MeRy-B (http://services.cbib.u-bordeaux.fr/MERYB/), HMDB (https://hmdb.ca/), and BMRB (http://www.bmrb.wisc.edu/)], spectra of reference compounds acquired in-house, and additional NMR experiments [Carr–Purcell–Meiboom–Gill (CPMG), TOtal Correlation SpectroscopY (TOCSY), Distortionless Enhancement by Polarization Transfer (DEPT135), COrrelation SpectroscopY (COSY), J-RESolved (JRES), Heteronuclear Single-Quantum Correlation spectroscopy (HSQC), and Heteronuclear Multiple-Bond Correlation spectroscopy (HMBC)]. The one-dimensional (1D) ^1^H-NMR spectra were processed with NMRProcFlow (https://nmrprocflow.org/; Jacob *et al.*, 2017). Metabolites were quantified using a 90 ° pulse calibration for acquisition, a glucose calibration curve, and the proton amounts of selected resonances. 

Targeted robotized microplate assays were used to quantify major metabolic traits. Following ethanolic fractionation of 20 mg fresh weight (FW) fruit tissue based on Sonnewald *et al.* (1991), glucose, fructose, and sucrose (Stitt *et al.*, 1989), sorbitol (Desnoues *et al.*, 2014), citrate (Tompkins and Toffaletti, 1982), malate (Möllering, 1974), and total free amino acids (Bantan-Polak *et al.*, 2001) were determined in the supernatant, and total soluble proteins (Bradford, 1976) and starch (Hendriks *et al.*, 2003) were determined in the pellet. For the analysis of ascorbate, fresh frozen powder was extracted with phosphoric acid (5%, v/v), and the supernatant was used immediately for the ascorbate assay (Gillespie and Ainsworth, 2007).

To estimate fruit lipid contents, fatty acid methyl esters (FAMEs) were measured after hydrolysis of 20 mg FW fruit tissue with 2.5% H_2_SO_4_ (v/v) in methanol. Gas chromatography with flame ionization detection (GC-FID) analysis was performed using an Agilent 7890 gas chromatograph (Agilent, Santa Clara, CA, USA) equipped with a DB-23 column (60 m × 0.25 mm, 0.25 μm; Agilent), a gas flow of 1.9 ml min^–1^_,_ and flame ionization detection. The temperature gradient was 50 °C for 1 min, increased to 175 °C at 25 °C min^–1^ and then to 230 °C at 2 °C min^–1^. FAMEs were identified by comparing their retention times with commercial fatty acid standards (Sigma, Saint-Quentin Fallavier, France) and quantified using a C17:0 internal standard and ChemStation (Agilent).

Fruit cell-wall polysaccharides were characterized according to Foster *et al.* (2010). Alcohol-insoluble residues (AIR) were isolated by washing 50 mg of lyophilized powder successively with 1.5 ml ethanol 70% (v/v), 1.5 ml chloroform/methanol (1:1; v/v) four times, and 500 µl acetone. After each washing step, samples were vortexed and centrifuged (16 000 *g*, 5 min) and the supernatant was removed. To determine the composition of non-cellulosic matrix polysaccharides (Jablonowski *et al.*, 2017), approximately 2 mg AIR was hydrolysed by adding 250 µl 2 M trifluoroacetic acid (TFA) for 90 min at 121 °C. A 100 µl aliquot of the TFA hydrolysate was evaporated under air flow and the dried pellet was dissolved in 400 µl of pure water. Monosaccharides were quantified using a high-performance anion exchange chromatography system coupled with pulsed amperometric detection (HPAE-PAD; Dionex system equipped with a CarboPac PA20 column and GP50, ED50, and AS50 modules). The column operating at a constant flow rate of 0.5 ml min^–1^ was equilibrated with 2 mM NaOH for 10 min before sample injection. Neutral monosaccharides were eluted with 2 mM NaOH over the course of 29 min, followed by 550 mM NaOH for 7.5 min to elute uronic acids. Subsequently, the column was rinsed with 800 mM NaOH for 5 min. Each monosaccharide amount was normalized to the internal standard (2-deoxy-D-glucose) and quantified using a specific calibration curve. Values for non-cellulosic glucose were obtained after subtracting the starch-glucose content.

Crystalline cellulose content was determined as described by Foster *et al.* (2010) with minor modifications. Dry AIR (2 mg) was hydrolysed with 1 ml of Updegraff reagent (Updegraff, 1969) at 100 °C for 30 min. The remaining residues were rinsed three times with pure water, then dried under air flow at 30 °C and hydrolysed using 175 µl of 72% (v/v) sulfuric acid for 45 min at room temperature, and then diluted with 925 µl of pure water. The amount of glucose released was quantified by an anthrone assay with a glucose calibration curve (Foster *et al.*, 2010).

### Fruit growth modelling and statistical analyses of compositional data

Fruit growth was modelled by fitting log-transformed FW data with sigmoid or double sigmoid functions and by using least squares to select the best fits. Growth fit (*y*(*x*)) parameters were then used to calculate growth rate (*y’*(*x*)) and RGR (*y’*(*x*)/*y*(*x*)).

To calculate starch net accumulation rates, starch content, log-expressed on a per fruit basis, was fitted by polynomial regression to calculate rates by derivation. To compare all fruits, the accumulation rate was then divided by fruit FW and expressed in mmol eq. glucose g^–1^ FW day^–1^.

Before computing correlations and multilinear regressions on mean data, 247 missing data were predicted using linear modelling over the total of 2478. Means of biological replicates of compositional data for each stage were visualized using a heatmap, after normalization (*z*-scores) and then hierarchical clustering (average linkage, Pearson distance) of the data in TM4 (Saeed *et al.*, 2003).

Generalized multilinear models were built in R using the glmnet package (Friedman *et al.*, 2010) to associate RGR and compositional data. Internal cross-validation was used to create the models. Random sampling of 100 000 models of RGR was performed to assess the likelihood of overfitting. The hdi package (Dezeure *et al.*, 2015) was used to test the statistical significance of each variable. To study the relative importance of each metabolite in the models, contents were multiplied by the weights associated with the different variables. To perform RGR predictions, data of each combination of seven species were used to build models, and data obtained for the eighth species were used to validate them. For this, datasets used to build a given model were randomly split into two subsets, one to build (80% of data) and one to cross-validate (20% of data). Each best-performing model was then used to predict RGR for the eighth fruit. For each of the eight species, 100 models were constructed to assess the stability of the prediction.

Partial least-squares discriminant analyses (PLS-DA) or orthogonal signal correction partial least-squares discriminant analyses (OSC-PLS-DA) with two orthogonal signal correction filtrations (OSC2-PLS-DA) were performed with BioStatFlow (http://biostatflow.org) to discriminate groups of species based on composition. Missing data on replicates were imputed by the NIPALS (Nonlinear Iterative PArtial Least Squares) algorithm (Wold, 1975). Then, five developmental stages were defined: A (maximum RGR/25), B (maximum RGR/2), C (maximum RGR), D (onset of ripening), and E (ripeness). Stage D was defined according to the fruit skin colour change occurring just before the end of growth in eggplant, clementine, and peach, to green commercial maturity when the fruit had reached its maximum size just before the colour change in pepper, to growth dropping in cucumber, to veraison in grapes, and to growth arrest and the beginning of starch degradation in apple and kiwifruit. Stage E corresponded to physiological or commercial maturity (see Supplementary Table S3 at *JXB* online). Discriminant compounds were highlighted based on the variable importance in the projection (VIP) score values. A two-factor (stage and species type) analysis of variance (ANOVA) combined with a false discovery rate (FDR) correction (Vinaixa *et al.*, 2012) was used to highlight the effect of sample groups and stage of development. Means of variables highlighted with multivariate analysis or ANOVA were compared using a mean comparison test for different fruit categories (herbaceous, vine, and tree species, or climacteric and non-climacteric species).

## Results

Eight species of fleshy fruits were compared throughout fruit development. They included three herbaceous species (eggplant, pepper, and cucumber), three tree species (apple, peach, and clementine), and two vines (kiwifruit and grape). Apple, peach, and kiwifruit are climacteric species.

### Sigmoid or double sigmoid functions provide the best fits for fruit growth

**Fig. 1.**
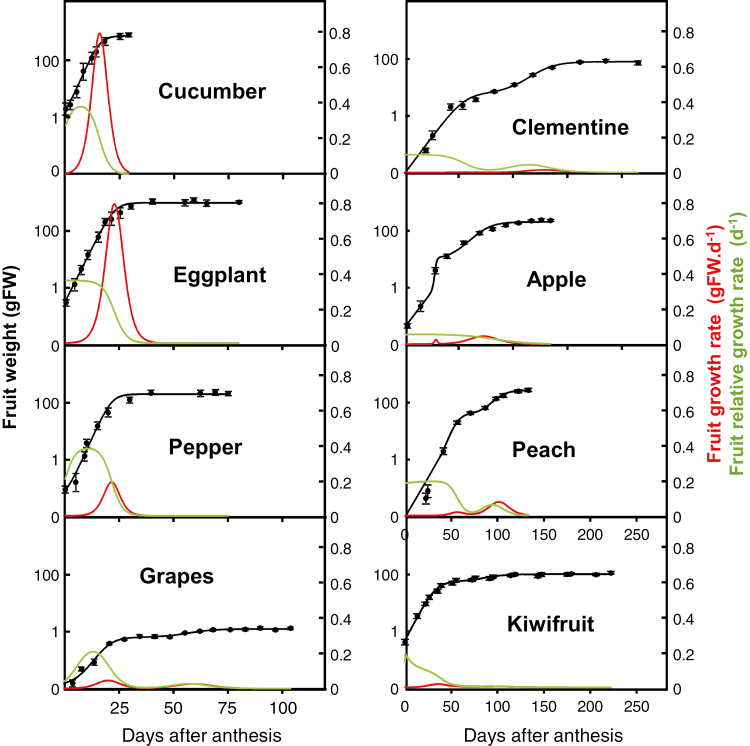
Growth of eight fleshy fruit species. Fruit fresh weight (black dots, means ±SD of *n*=20 for the three earliest stages and *n*=10 for other stages of development) and growth fittings (black lines), which are sigmoid functions for cucumber, eggplant, and pepper, and double sigmoid functions for eggplant, grape, apple, peach, and kiwifruit, on a log scale. Fruit growth rates are represented in red and fruit relative growth rates in green.

The duration of fruit development from anthesis to maturity was highly variable between species (Table 1, Fig. 1), ranging from 29 DAA in cucumber (which corresponds to commercial maturity rather than physiological maturity for this species) to 253 DAA for clementine. A simple sigmoid (eggplant, pepper, and cucumber) or double sigmoid (apple, peach, kiwifruit, clementine, and grape berry) gave the best fits to model growth, which was plotted on a logarithmic axis to maximize accuracy for the youngest fruits. Calculated growth rates varied dramatically between fruit species (Fig. 1; Table 1), with the highest values for the largest fruit (cucumber and eggplant) and the lowest values for the smallest (grape; Fig. 1, Table 1).

**Table 1.** Growth and development of eight fleshy fruit species

	Eggplant	Pepper	Apple	Peach	Cucumber	Clementine	Kiwifruit	Grape
Duration of development (DAA)	80	76	157	133	29	**253**	222	110
Duration of growth (DAA)	40	40	136	133	25	**218**	147	77
Maximum weight (g)	**1135**	232	218	276	864	87	106	1.5
Maximum growth rate (g day^–1^)	79	19	4.4	7.8	**80**	1.4	1.9	0.038
Maximum relative growth rate (g g^–1^ day^–1^)	0.354	0.379	0.054	0.194	**0.383**	0.100	0.189	0.207

Duration of development corresponds to the time between anthesis and maturity; duration of growth corresponds to the time during which the growth rate was >0; maximum weight was obtained by averaging values obtained for at least eight fruits; maximum growth rate and relative growth rate were calculated as detailed in the Materials and methods. Highest values for each variable are in bold text. DAA, Days after anthesis.

As expected, RGR (expressed in day^–1^) presented one or two peaks for fruits whose growth followed a single or double sigmoid function, respectively (Fig. 1). In the latter case, the second peak was always lower than the first. In the herbaceous species (cucumber, eggplant, and pepper), RGR peaked at the beginning of fruit development, whereas in the tree species (clementine, apple, and peach) RGR decreased after an initial plateau. The highest maximum RGR was found in cucumber, closely followed by pepper and eggplant. Grape, peach, and kiwifruit showed intermediate values. Clementine and apple showed the lowest maximum RGR.

### Changes in biomass composition throughout fruit development

The next step in comparing the eight fruit species consisted of identifying the maximum number of common and detectable metabolic traits in an unbiased way. For this, a biological standard, obtained by mixing equal amounts of samples from all developmental stages, was prepared for each species and then analysed by several techniques. NMR spectroscopy was used to identify (Supplementary Table S4, Supplementary Fig. S1) and then quantify (Supplementary Table S5) the most abundant polar soluble compounds, that is, metabolites that constitute more than 0.1 mg g^–1^ FW according to the detection method (Moing *et al.*, 2004). In all species, sucrose, fructose, and glucose were by far the most abundant detected sugars. Malate and citrate were always among the most abundant organic acids, but quinate and tartrate were more abundant in kiwifruit and grape, respectively. However, quinate was not detected in all species and tartrate was detected only in grape, as expected (Debolt *et al.*, 2006). Individual amino acids had very variable abundances or were not always detected. We therefore decided to measure total free amino acids instead. Relatively abundant metabolites belonging to other classes (i.e. tyramine, dimethyl proline, and synephrine) were detected only in clementine. Subsequently, sucrose, glucose, fructose, malate, citrate, and total amino acids were detected in all species and at all fruit developmental stages using targeted microplate methods, and reduced and oxidized ascorbate were also analysed. The major fatty acids analysed after esterification of apolar extracts were identified and quantified by GC-FID; the most abundant were palmitate (C16:0), stearate (C18:0), oleate (C18:1 *cis*-9), and linoleate (C18:2 *cis*-9,12), and linolenate (C18:3 *cis*-9,12,15), arachidate (C20:0), and lignocerate (C24:0) were also detected. With regard to sugar polymers, starch, crystalline cellulose, and the major constituents of the cell-wall polysaccharides (the monosaccharide derivatives fucose, xylose, arabinose, galactose, mannose, rhamnose, glucose, galacturonate, and glucuronate) were also found and quantified in all species (Supplementary Table S6).

**Fig. 2.**
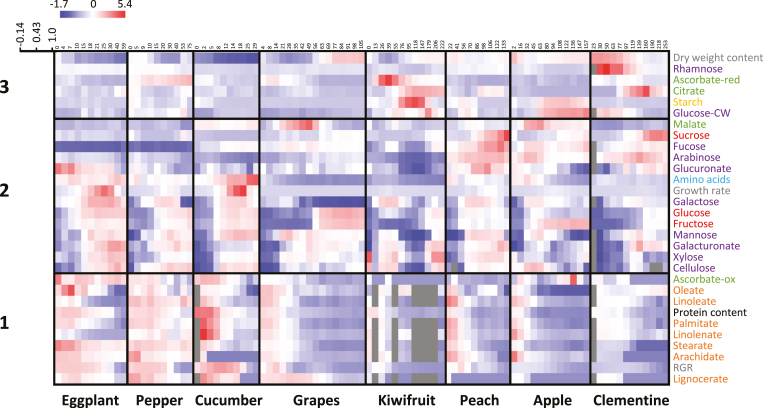
Overview of changes in biomass composition during fruit development of eight species. Heat maps based on *z*-scores of mean values per stage (*n*=4 or 5) of contents expressed on a dry weight basis. Columns correspond to stages of development and rows correspond to clustered contents of 28 common compounds, as well as growth rate and relative growth rate (RGR) (based on Pearson correlation coefficient). Grey corresponds to missing data.

To obtain an overview of the changes in biomass composition during fruit development, clustering analysis was performed for compositional and growth data (Fig. 2). The first major cluster (Cluster 1) contained all fatty acids, protein, and oxidized ascorbate. These variables, which were also correlated with RGR, were highest in young stages of development and in herbaceous fruits. Cluster 2 contained most cell-wall components, major soluble sugars, soluble amino acids, and malate, as well as growth rate. Most of these metabolic variables increased in the later stages of fruit development and/or at ripening. Cluster 3 contained fewer metabolic traits (e.g. starch, citrate, reduced ascorbate) that were less strongly correlated with each other. Taken together, these results indicate that changes in a range of major biomass components during fruit development are similar among very diverse species, and that links with other traits, such as RGR, may be established. Studies have shown that within species, plant performance traits such as shoot biomass and yield can be predicted from metabolic composition by using genetic diversity panels (e.g. Meyer *et al.*, 2007; Riedelsheimer *et al.*, 2012).

### Relative growth rate can be predicted from biomass composition

As shown in Fig. 2, RGR appeared to be well correlated with several metabolic variables. Indeed, 11 of the 28 measured metabolic variables common to the eight fruit species and expressed on a dry weight (DW) basis were significantly (*P*<0.05 after Bonferroni correction) correlated with RGR. The most significant correlation was found for total protein content (*R*=0.84, *P*=0), then for stearate (*R*=0.72, *P*=9.4.10^–13^.), palmitate (*R*=0.72, *P*=7.4.10^–13^), and lignocerate (*R*=0.68, *P*=5.6.10^–11^), suggesting that high RGR involves high protein content and the production of stearate-, palmitate-, and lignocerate-rich membranes.

**Fig. 3.**
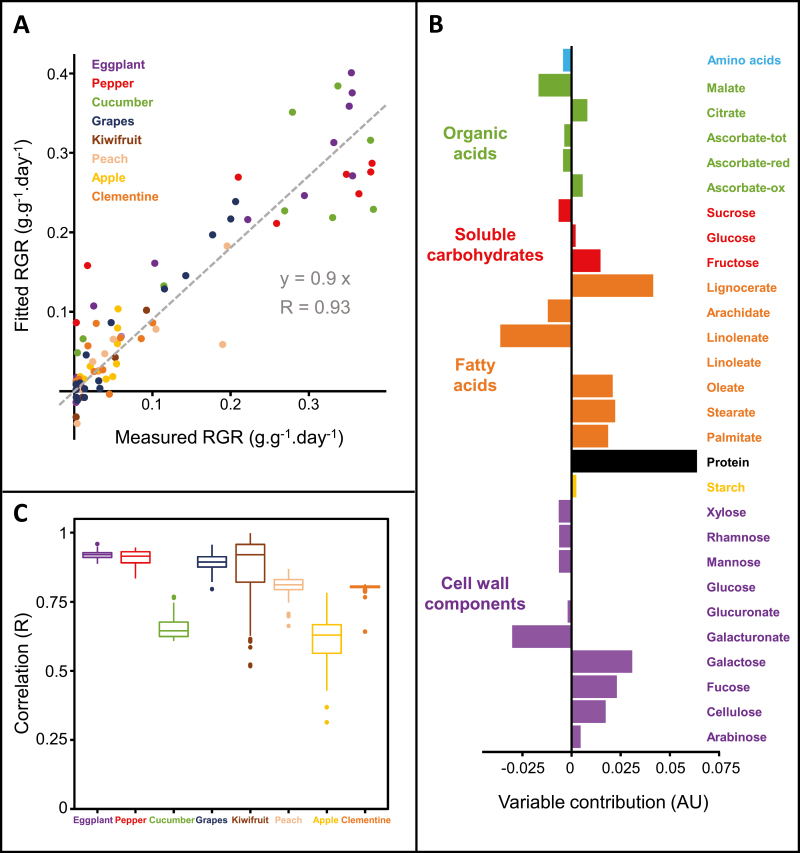
Generalized linear modelling for the prediction of relative growth rate (RGR) from contents of 28 compounds quantified in eight fruit species and at all developmental stages. (A) Correlation graph between RGR values measured and calculated by a model for all fruits from concentrations expressed per g dry matter. (B) Importance of the contribution of variables classified by biochemical family for the linear model. (C) Box plot representing the distribution of correlation coefficients (*n*=100) obtained for each fruit species by using models constructed with data obtained from the other seven species.

A generalized linear modelling approach (Friedman *et al.*, 2010) was then used to predict RGR from biomass composition for all fruits and stages of development. RGR values calculated by the model (Fig. 3A) were highly correlated with the experimental values (*R*=0.93), confirming that fruit RGR is strongly related to biomass composition. To check the likelihood of a good fit, 100 000 models were created by randomly assigning the measured RGR to the different fruits and stages of development. The model obtained with the real RGR was clearly the best among the 100 000, with the second best model having a correlation of *R*=0.82. Three cell wall constituents, four fatty acids, and protein content contributed the most to the model (Fig. 3B). A bootstrap approach showed that protein content, which was associated with higher RGR, was statistically significant in the model (*P*=1.4.10^–10^). Cell wall-derived galacturonate, galactose, lignocerate, and linolenate were important variables associated with lower RGR, but were not statistically validated by the bootstrap approach, meaning that they are more likely to have a different effect on different fruits. To evaluate whether our predictions were valid, we then built 100 models for each species using the data from the seven other species. In all cases, the model correctly predicted the RGR (Fig. 3C). Published data for growth and proteins (Biais *et al.*, 2014) and cell-wall-derived galactose (Colombié *et al.*, 2015) measured in developing tomato fruit further validated this result (Supplementary Fig. S3), with significant Pearson correlations between RGR and galactose (*P=*0.0003) and between RGR and protein content (*P*=0.027).

### Fruits of herbaceous plants, vines, and trees are distinguishable based on biomass composition

**Fig. 4.**
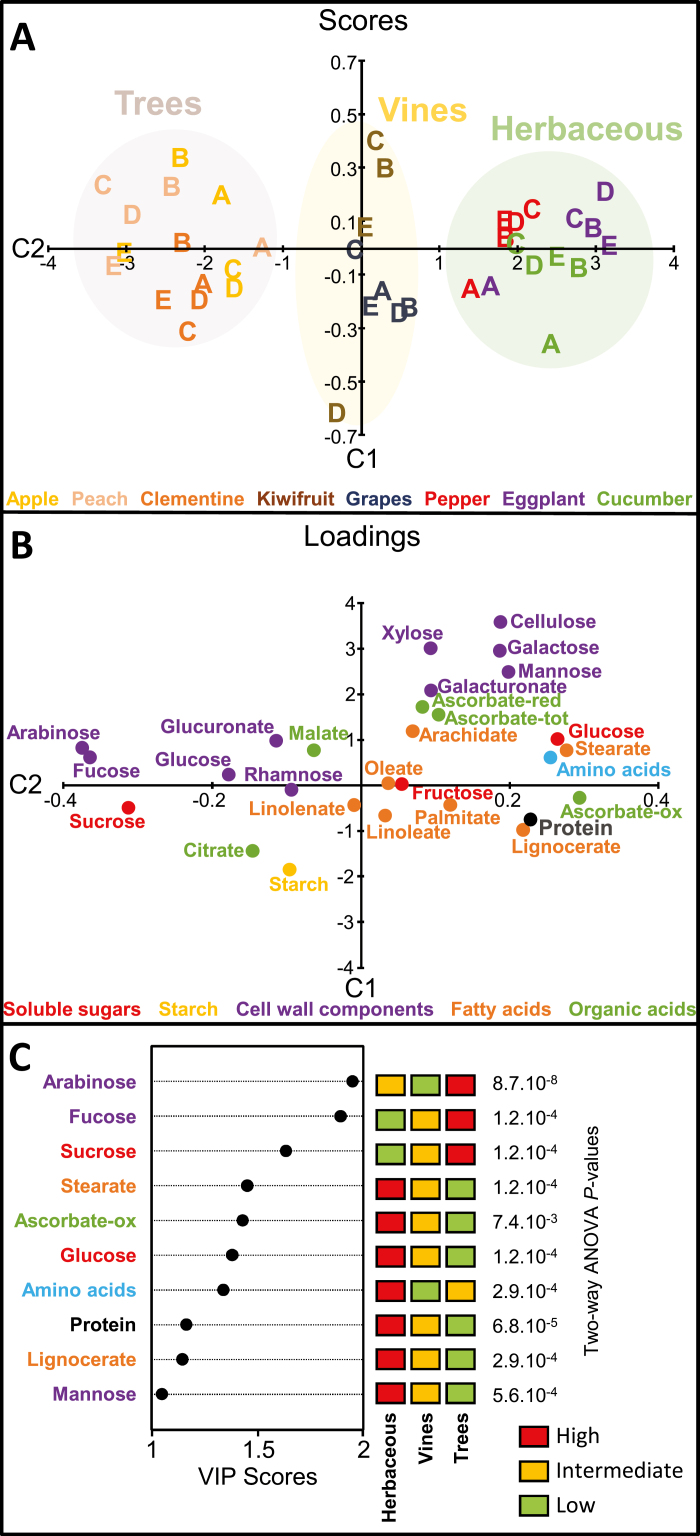
Discriminant analysis of herbaceous, vine, and tree species. OSC2-PLS-DA was performed with 28 variables (compound mean values per stage) measured in 39 samples corresponding to four (for kiwifruit) or five (for all other species) selected stages of development in eight species of fleshy fruits. (A) Scores plot on the first two components. (B) Loadings plot on the first two components. The compounds are coloured according to their biochemical family (see also Fig. 3B). Model quality parameters: R^2^Y=94%, Q^2^=0.93, *P*=0.015. (C) Representation of the variables with VIP scores >1 for the OSC2-PLS-DA model. The *P*-value of two-way ANOVA with FDR correction is shown on the right for each compound.

A multivariate discriminant analysis (OSC-PLS-DA) was performed to separate herbaceous species (eggplant, pepper, and cucumber), vines (kiwifruit and grape), and trees (apple, peach, and clementine) based on their fruit compositional data on a DW basis (Fig. 4). Two orthogonal signal correction (OSC) filtrations were necessary to clearly separate the three groups of species. The root mean-square error of prediction (RMSEP) was 0.28 and the prediction quality of the model (Q^2^ predictive variance, calculated by cross-validation) was 0.93. The first component (C1, latent variable), which explained 94% of the variance of Y (species) and 25% of the variance of the matrix X of the 28 compounds (Fig. 4A, B), distinguished herbaceous species from vine and tree species for all stages of development. The three compounds with the highest VIP scores (Fig. 4C), which tended to be more abundant in fruits of tree species, were two components of cell-wall polymers (arabinose and fucose) and sucrose. Stearate, oxidized ascorbate, total free amino acids, and free glucose had VIP scores between 1.2 and 1.6 and were higher in herbaceous species. Two-way ANOVAs were performed for all compounds for stage and herbaceous type effects and their interaction. The *P*-values for herbaceous type effect of variables with VIP scores higher than 1 are presented in Fig. 4C.

All these variables were significantly affected by herbaceous type effect, and the lowest two *P*-values were observed for cell wall arabinose and total proteins. As a first validation step, we used published data for arabinose (Gross and Sams, 1984) and protein content (USDA food database, https://ndb.nal.usda.gov/ndb/nutrients/index). For arabinose, with the data from three stages of fruit maturation for 17 species, including 13 species not considered in the present study, we performed a two-way ANOVA to compare tree, vine, and herbaceous species. The ANOVA *P*-value for the herbaceous type effect was significant (*P*=5.10^–8^). A mean comparison test was performed on the same data, with mean values of 87, 19, and 24 mg arabinose per 100 mg cell wall for tree, vine, and herbaceous species, respectively. The fruits of herbaceous species differed significantly from those of trees (Tukey’s studentized test, *P*=4.10^–8^). For protein content, data corresponding to 57 tree, 6 vine, and 24 herbaceous species at fruit maturity (see Supplementary Table S7) were expressed on a DW basis, then used to perform a one-way ANOVA for herbaceous type. The ANOVA *P*-value for the herbaceous type effect was 1.10^–7^, with mean values of total proteins of 11.2, 7.4, and 5.8 g 100 g^–1^ DW for herbaceous, vine, and tree species, respectively. A mean comparison test showed that the fruit protein content of herbaceous species differed significantly from that of tree species (Tukey’s studentized test, *P*=2.10^–8^), in agreement with the experimental data from the present study for eight species during development. Vines also differed significantly from herbaceous species based on the USDA data on protein content in fruit (Tukey’s studentized test, *P*=0.02), but not from tree species.

### Biomass composition differentiates climacteric from non-climacteric fruits

**Fig. 5.**
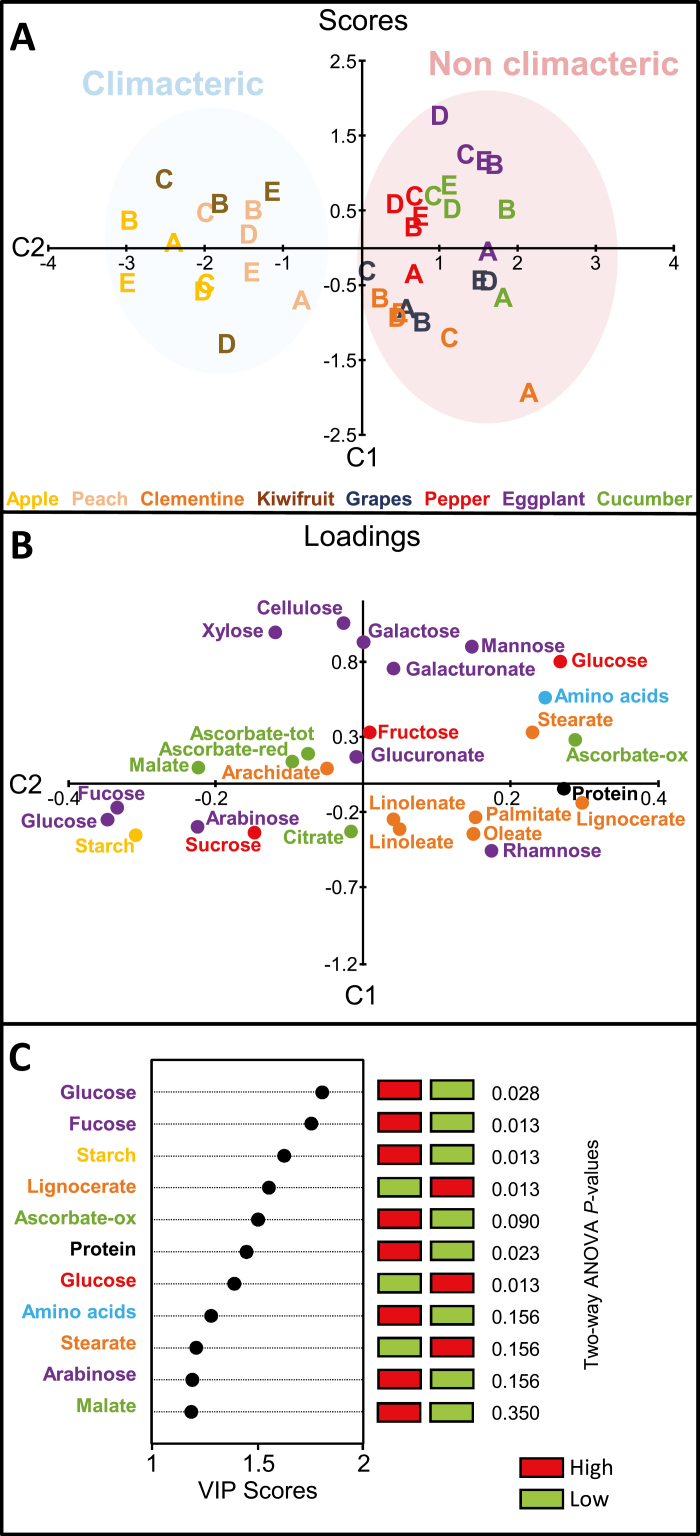
Discriminant analysis of three climacteric and five non-climacteric fruit species. OSC2-PLS-DA was performed with 28 variables (compound mean values per stage) measured in 39 samples corresponding to four (for kiwifruit) or five (for all other species selected stages of development in eight species of fleshy fruits. (A) Scores plot on the first two components. (B) Loadings plot on the first two components. Compounds are coloured according to their biochemical family (see also Fig. 3B). Model quality parameters: R^2^Y=86%, Q^2^=0.89, *P*=0.003. (C) Representation of variables with VIP scores >1 for the OSC2-PLS-DA model. The *P*-value of two-way ANOVA with FDR correction is shown on the right for each compound.

A second PLS discriminant analysis was used to differentiate climacteric (apple, peach, and kiwifruit) and non-climacteric (eggplant, pepper, cucumber, clementine, and grape) fruit (Fig 5A, B). Two OSC filtering steps were necessary to develop a classification model with a good separation of the two groups along C1. RMSEP was 0.26 and Q^2^ was 0.89. As shown in Fig. 5A, C1 explained 86% of the variance of Y (climacteric versus non-climacteric) and 15% of the variance of matrix X of the 28 compounds. Samples from non-climacteric fruit (on the positive side of C1) were easily distinguished from those of climacteric fruit (on the negative side of C1) at all developmental stages. The highest VIP score was 1.80 for cell-wall glucose, which tended to be higher in climacteric fruit (Fig. 5C). The other two highest VIP scores (>1.6) were found for cell-wall fucose and starch, which were also higher in climacteric fruit. In contrast, lignocerate, stearate, total protein, oxidized ascorbate, amino acids, and free glucose had VIP scores between 1.6 and 1.2, and tended to be more abundant in non-climacteric fruit. Two-way ANOVAs were performed for all compounds for stage and climacteric type effects and their interaction (Fig 5C). Six of these variables were significantly affected by climacteric type effect (starch, lignocerate, free glucose, cell-wall fucose, cell-wall glucose, and total proteins). The lowest two *P*-values were observed for starch and lignocerate. Because of the overrepresentation of herbaceous species in the non-climacteric group, an additional OSC-PLS-DA analysis was performed with tree and vine species only (Supplementary Fig. S2). Among variables with VIP scores greater than 1, cell-wall glucose and fucose, as well as starch, again tended to be higher in climacteric fruit, whereas lignocerate and free amino acids again tended to be higher in non-climacteric fruit. A two-way ANOVA performed for each of these five variables showed that all were significantly different, except for total amino acids.

**Fig. 6.**
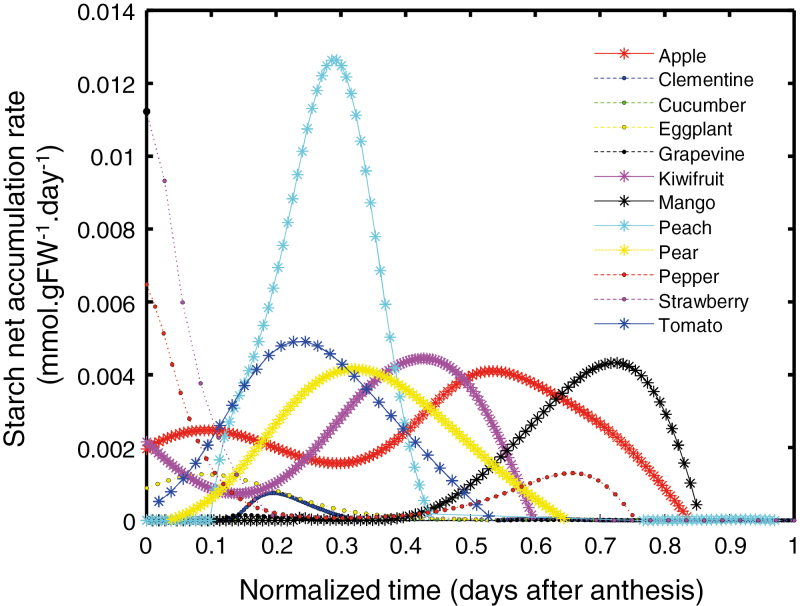
Starch net accumulation rates in 12 fleshy fruit species. Rates (expressed in mmol g^–1^ FW day^–1^ of glucose equivalent) were calculated from the fit of starch accumulation and fruit growth. Additional datasets were collected for tomato (Biais *et al.*, 2014), strawberry (Cakpo *et al.*, 2020), mango (Tandon and Kalra, 1983), and pear (Oikawa *et al.*, 2015) fruits. Time is normalized against total time from anthesis to ripeness. Climacteric species are indicated with asterisks and solid lines.

Starch appeared to be of central importance in climacteric fruits, so we compared starch accumulation rates between fruits of all eight species (Fig. 6), plus four species for which published data were available: tomato (Biais *et al.*, 2014), strawberry (Cakpo *et al.*, 2020), mango (Tandon and Kalra, 1983), and pear (Oikawa *et al.*, 2015). The calculated starch net accumulation rate appeared to be higher for climacteric fruits than for non-climacteric fruits, with peach displaying the highest rate (Fig. 6). For the non-climacteric species strawberry and pepper, high starch accumulation rates were also evident, but they peaked before anthesis. Pepper had a relatively high rate of starch accumulation during late growth, but it was lower than for climacteric fruits.

## Discussion

The present study compared a panel of species during their development by quantitative analyses of the composition of their fruit. Therefore, we needed quantitative data for a relatively large and diverse spectrum of biomass components that are common to these species and had to standardize the stages of fruit development to facilitate comparison.

### Data standardization to enable comparison of fruit species

Although many studies have been conducted on the composition of fruits during their development or maturation (e.g. Nielsen *et al.*, 1991, Marsh *et al.*, 1999, Zhang *et al.*, 2010, Lombardo *et al.*, 2011, Dai *et al.*, 2013, Nardozza *et al.*, 2013, Biais *et al.*, 2014), only a few comparative studies of species have been performed in recent years (e.g. Osorio *et al.*, 2012; Bae *et al.*, 2014; Klie *et al.*, 2014; Dai *et al.*, 2016). The main difficulty with such comparisons is the heterogeneity of the results obtained for a given metabolic trait by different laboratories that use different procedures and instrumentation (Quentin *et al.*, 2015). In the present study, harvests were carried out in the same year and all the quantitative analyses were performed in the same period using the same methods of extraction and analysis, and the same instrumentation. Together, these factors allowed a closer comparison between species.

Another challenge for multi-species comparisons is that not only stages and durations of fruit development but also visual phenotypes are generally different among species (Schauer *et al.*, 2005). Modelling fruit growth made it possible to classify and select stages of development according to growth rate and phenotypic data. Modelling was done on a daily basis because the cardinal temperature values (base, optimal limits, and maximum temperatures) for growth, for example, for tomato (Boote *et al.*, 2012), are not available for all the species studied here.

### Fruits of herbaceous species grow faster than tree and vine fruits

The fruits of the herbaceous species reached their maximum weight rapidly and had high RGRs, up to nearly 40% FW per day for cucumber (Table 1). Conversely, the fruits of the woody (i.e. tree and vine) species showed lower RGRs, in agreement with published data, for example, for pear, in which RGRs of ~6% day^–1^ were recorded at 40 days after flowering (Shiratake *et al.*, 1997), or cherry fruit, with 20% day^–1^ at 69–95 days between anthesis and maturity (Gibeaut *et al.*, 2017). Woody species also showed much longer growth periods. Thus, clementine had a particularly low maximum RGR (10% day^–1^) and needed more than 200 days to develop and mature. Finally, the fruits of both vine species had a maximum RGR and a time to reach maximum RGR that were intermediate, but a relatively long maturation period. RGR data for early stages of fruit development remain scarce in the literature, but the fastest-growing fruits are undoubtedly herbaceous; for example, melon (*Cucumis melo*) fruit has an RGR reaching 80% day^–1^ for a total duration of development of 40–50 days (Gao *et al.*, 1999). Two hypotheses might explain such differences in growth rate between herbaceous and woody species.

First, it is tempting to see the modulation of growth rate as a consequence of source–sink relationships, the main principle of the theory of reproduction costs being that there is a trade-off between allocation to vegetative growth and allocation to reproduction (Obeso, 2002). In general, fast-growing species produce relatively more leaf area and less root mass (Lambers and Poorter, 1992), which would tend to limit non-reproductive sink strength. In addition, slow-growing species such as trees invest a large proportion of energy in the vegetative biomass of foliage and perennial structures, which increases the competition between reproductive and non-reproductive sinks. Thus, in peach, for example, the vegetative biomass represents more than 50% of the energy costs, of which approximately 30% is for wood (Berman and Dejong, 2003). However, many studies have shown that woody plants have high concentrations of carbon and other nutrients in vascular tissues, suggesting that fruit growth is not more limited in carbon than it is in herbaceous plants (Martinez‐Vilalta *et al*., 2016; Tixier *et al.*, 2018). In addition, although the culture conditions used here were intended to maximize nutrient supply to the fruit, it is accepted that the production of herbaceous fruit, even under production conditions, is also limited by the source (Li *et al.*, 2015). Moreover, several studies have shown that when the number of fruits per plant is decreased, larger fruits are obtained (e.g. Link, 2000; Webster and Spencer, 2000). However, such spectacular effects on fruit size do not require large changes in RGR. For example, a study on grape berry showed that a change of less than 40% of RGR during the first third of fruit development led to a change of ~100% in mature fruit FW (Ollat and Gaudillère, 1998). It is therefore likely that source–sink relationships influence RGR only marginally.

The second hypothesis is that the growth rate of the fruit depends on the organization and composition of its biomass. This is suggested by the close relationship found here between the composition of certain components of the fruit biomass and RGR. It should be noted that the resulting linear model works for both the different developmental stages and species. The compounds involved in this relationship were essentially distributed between lipids, cell walls, and proteins, that is, structural components of the biomass.

Cell-wall galacturonate, rhamnose, mannose, and xylose contributed negatively to RGR, which means that these compounds tended to be more abundant in the cell wall of slow-growing or slow-ripening fruits (Fig. 3). The contents of two of these compounds may reflect the amount of pectin, since pectin is essentially composed of galacturonate polymers but also has relatively abundant rhamnose (rhamnogalacturonan) residues (Garleb *et al.*, 1991). Minor amounts of xylose are also found in the form of xylogalacturonan in pectin (Mohnen, 2008), but it is a major constituent of xyloglucans and xylans, which are hemicellulosic glycans (Scheller and Ulvskov, 2010). In primary cell walls, xylans can bind pectin (Broxtermann and Schols, 2018; Tucker *et al.*, 2018). The role of xyloglucans in wall structure and wall extensibility has been revised in recent years and they are now thought to be important for the biomechanics of growing cell walls (Park and Cosgrove, 2015). Galactose is the building block of neutral side chains in pectins (β-galactans) (Atmodjo *et al.*, 2013) but it is also part of xyloglucan and galactoglucomannan (Pauly *et al.*, 2013). Whereas mannose is the major building block of mannans, galactans are connected with stiffer walls and are degraded during fruit softening (Ross *et al.*, 1993; Wefers *et al.*, 2018). Mannans, which also provide mechanical support, are modified during ripening (Rodríguez-Gacio *et al.*, 2012; Tucker *et al.*, 2018). Taken together, these results suggest that more polysaccharides that are important for cell-wall stiffness are produced in fast-growing fruit, whereas when RGR is lower, it is especially the pectin backbone that increases, thus rendering the wall thicker and less extensible.

With regard to lipids, the lignocerate content was strongly correlated with RGR. Surprisingly, there is little information on lignocerate in the plant literature. However, the application of lignocerate to cotton ovules was shown to stimulate their growth via ethylene (Qin *et al.*, 2007), stimulating in particular the synthesis of walls (Pang *et al.*, 2010). Furthermore, salt stress, which inhibited growth, led to a sharp decrease in lignocerate in the leaves of *Artemisia annua* (Qureshi *et al.*, 2013).

Finally, the highest protein contents were found in the young fruits of herbaceous plants, followed by the fruits in which there is a resumption of growth during their development (grape, kiwifruit, and peach). The correlation with RGR appeared to be particularly high. Although such a relationship has not been reported for fruits, a weak but significant correlation was found for leaves between protein content, estimated by pyrolysis-mass spectrometry, and RGR in panels of 11 grasses (Niemann *et al.*, 1992) and 24 herbaceous species (Niemann *et al.*, 1995). Moreover, a study in which the Col-0 accession of Arabidopsis was grown under different photoperiods showed a strong (*R*=0.9) correlation between total soluble protein content and RGR (Gibon *et al.*, 2009). A simple interpretation for these observations is that growth depends on the amount of catalysts, that is, proteins. This interpretation is in line with the ‘growth rate hypothesis’, according to which growth is limited by the capacity for protein synthesis and especially the concentration of ribosomes, since the latter controls protein translation when limiting (Elser *et al.*, 2000; Giordano *et al.*, 2015). However, this hypothesis has been challenged. For example, a negative correlation between growth rate and protein content was shown in a panel of Arabidopsis accessions (Ishihara *et al.*, 2017), with the authors attributing this observation to the energy cost of protein turnover. They posited that the accessions that produce the most leaf biomass have lower ribosome abundance and lower protein synthesis but a lower rate of protein degradation, which would increase the efficiency of growth by optimizing the investment in the photosynthetic apparatus.

### Climacteric character is established well before fruit ripening

The distinction between climacteric and non-climacteric fruit has been based on how the fruit matures, with the respiratory crisis and autocatalytic production of ethylene characterizing climacteric fruit (Paul *et al.*, 2012). A recent study also highlighted three types of transcriptional feedback circuits controlling fruit maturation orchestrated by ethylene (Lü *et al.*, 2018). The present results suggest that the climacteric character is also linked to the composition of the fruit biomass.

OSC-PLS-DA showed that climacteric fruits are richer in certain types of cell-wall polysaccharides (estimated by their monomers) and starch, while non-climacteric fruits have higher contents of given lipids (determined as FAMEs), proteins, free amino acids, free glucose, and oxidized ascorbate (Fig. 5). Before the beginning of maturation, starch is indeed higher in kiwifruit, apple, pear, tomato, and banana, which are climacteric fruits (Okuse and Ryugo, 1981, Berüter, 1985, Cordenunsi and Lajolo, 1995, Biais *et al.*, 2014, Mesa *et al.*, 2016). In non-climacteric fruits such as eggplant (Makrogianni *et al.*, 2017), cucumber (Davies and Kempton, 1976), and strawberry (Moing *et al.*, 2001), starch content is very low or undetectable before the initiation of ripening. The contents of cell-wall glucose, arabinose, and fucose were generally higher in climacteric fruit (Fig. 5), especially apple and peach, in accordance with a previous study (Gross and Sams, 1984). This finding suggests that climacteric fruits have a greater accumulation of certain types of cell-wall polysaccharides and starch. These polymers might then be degraded massively during ripening, with part of the released energy being dissipated via non-phosphorylating oxidation (Colombié *et al.*, 2017). The observed high starch net synthesis rate for climacteric fruits (Fig. 6) suggests that starch accumulates with a specific granular structure and/or composition in fruits. For example, the concentrations of amylopectin, which corresponds to the branched structure of starch, and amylose, which corresponds to the linear chain, could be regulated specifically in climacteric fruits. If starch contains more amylopectin or if amylopectin is degraded before amylose, more than one glucose can be hydrolysed at the same time, thus increasing the energy produced. Unfortunately, data on amylopectin and amylose concentrations throughout fruit development are sparse. In apple, amylopectin was synthesized before amylose (Magein and Leurquin, 2000), and in banana, the amylopectin content in pulp increased more than the amylose content when total starch increased as the fruit developed (Miao *et al.*, 2017). These results indicate that the climacteric character may also be related to the nature of the compounds that accumulate during the growth phase of the fruit, and therefore well before the onset of the climacteric crisis.

In the present study, non-climacteric fruit had the highest protein and oxidized ascorbate contents throughout development (Fig. 5), possibly because the fruits studied here with the highest RGRs are non-climacteric. Ascorbate, which was relatively weakly associated with growth rate (see above), is a major antioxidant in fruit (Fenech *et al.*, 2019). Since an increased ratio of oxidized to reduced ascorbate reflects a greater flux of reactions between ascorbate and reactive oxygen species (Smirnoff, 2018), it is possible that starch accumulation and cell-wall synthesis, which were more prominent in climacteric fruit, are energy sinks capable of minimizing the production of reactive oxygen species. Furthermore, during maturation, climacteric fruit dissipates the excessive energy released following the degradation of these polymers (Colombié *et al.*, 2017).

## Conclusion

This comparative study of eight fruit species revealed a strong link between biomass composition and growth rate. We now need to search for similar links in other organs, such as leaves, and to target the biomass components that contribute the most to the model by classic or reverse genetic approaches in order to confirm their impact on growth rate. It will also be interesting to look for such a relationship within intra-species diversity panels and to assess the impact of the environment. Furthermore, the underlying trade-offs need to be elucidated; for example, whether it is possible to manipulate the total protein content by acting on protein synthesis, especially at the level of the ribosomes, without disturbing the construction of biomass or increasing the competition between sink organs. A better understanding of how biomass is built could lead to new strategies for improving plant performance.

We were able to discriminate between the main fruit types while highlighting the most relevant variables, especially structural components and the level of starch accumulation, that characterize climacteric fruit. While the action of ethylene during ripening is beginning to be well understood, questions remain regarding the mechanisms underlying the accumulation and degradation of these polymers. Understanding the mechanisms underlying polymer accumulation and degradation could lead to strategies aiming to obtain climacteric fruits in order to better control their maturation. For example, in the case of strawberry, this could greatly reduce the considerable losses that occur as a result of the very short time between harvest and over-maturity.

## Supplementary data

Supplementary data are available at *JXB* online.

**Fig. S1.** Annotated 1D ^1^H-NMR spectra of polar extracts of mixed-stage samples for each of eight fruit species.

**Fig. S2.** Discriminant analysis of three climacteric and five non-climacteric fruit species by OSC2-PLS-DA performed with 28 variables (compounds) in 39 observations corresponding to four or five selected stages of development.

**Fig. S3.** Correlation between cell-wall galactose or total soluble protein content and fruit RGR in tomato.

**Table S1.** Culture conditions for the eight fruit species.

**Table S2.** Growth model equations and parameters for fitting of the fresh weight of eight fruit species.

**Table S3.** Selected growth stages and corresponding DAA for comparison between fruit species.

**Table S4.** Summary of the NMR annotation of 1D ^1^H-NMR spectra of polar extract of mixed-stage samples of eight fruit species.

**Table S5.** Quantification of major compounds annotated from 1D ^1^H-NMR profiles of polar extract of mixed-stage samples of eight fruit species.

**Table S6**. Average content of major compounds in eight fruit species for 10–16 stages of development.

**Table S7**. Total protein data of 87 fruit species from the USDA food database (https://ndb.nal.usda.gov/ndb/nutrients/index).

**Table S8.** Average contents of major compounds in eight fruit species for 10–16 stages of development, expressed in percentage dry weight.

eraa302_suppl_Supplementary_Figures

eraa302_suppl_Supplementary_Tables

## Data Availability

All data used in this article are publicly available at https://data.inrae.fr/dataverse/frimouss. The ^1^H-NMR spectra have been deposited in the INRAE Dataverse repository (https://doi.org/10.15454/BQHGR9).
