# Supplementary material for: Biomass composition explains fruit relative growth rate and discriminates climacteric from non-climacteric species
Source: J Exp Bot. 2020 Jun 27;71(19):5823–36. doi: 10.1093/jxb/eraa302 (PMC7540837; doi:10.1093/jxb/eraa302)
Supplement: eraa302_suppl_Supplementary_Figures [file eraa302_suppl_supplementary_figures.pdf]

Supplementary Figures

Title: Biomass composition explains fruit growth rate and discriminates climacteric from non-climacteric species

Authors: Léa Roch[1], Sylvain Prigent[1], Holger Klose[2,3], Coffi Belmys Cakpo[4], Bertrand Beauvoit[1], Catherine Deborde[1,5], Laetitia Fouillen[5,6], Pierre van Delft[5,6], Daniel Jacob[1,5], Björn Usadel[2,3], Zhanwu Dai[7], Michel Génard[4], Gilles Vercambre[4], Sophie Colombié[1], Annick Moing[1,5], Yves Gibon[1,5]

[1]UMR 1332 Biologie du Fruit et Pathologie, INRA, Univ. Bordeaux, Centre INRA de Nouvelle Aquitaine - Bordeaux, av Edouard Bourlaux, 33140 Villenave d'Ornon, France

[2]Institute for Biology, RWTH Aachen University, Worringer Weg, 52074 Aachen, Germany

[3]Institute of Bio- and Geosciences, Plant Sciences (IBG-2), Forschungszentrum Jülich GmbH, Germany.

[4]UR 1115 PSH, INRA, F84914 Avignon Cedex 9, France

[5]Plateforme Métabolome du Centre de Génomique Fonctionnelle Bordeaux, MetaboHUB, IBVM, Centre INRA de Nouvelle Aquitaine - Bordeaux, av Edouard Bourlaux, 33140 Villenave d'Ornon, France

[6]UMR 5200, CNRS, Univ. Bordeaux, Laboratoire de Biogenèse Membranaire, av Edouard Bourlaux, 33140 Villenave d'Ornon, France

[7]UMR 1287 EGFV, INRA, Univ. Bordeaux, Bordeaux Sci Agro, F33883 Villenave d'Ornon, France

Corresponding author: yves.gibon@inra.fr

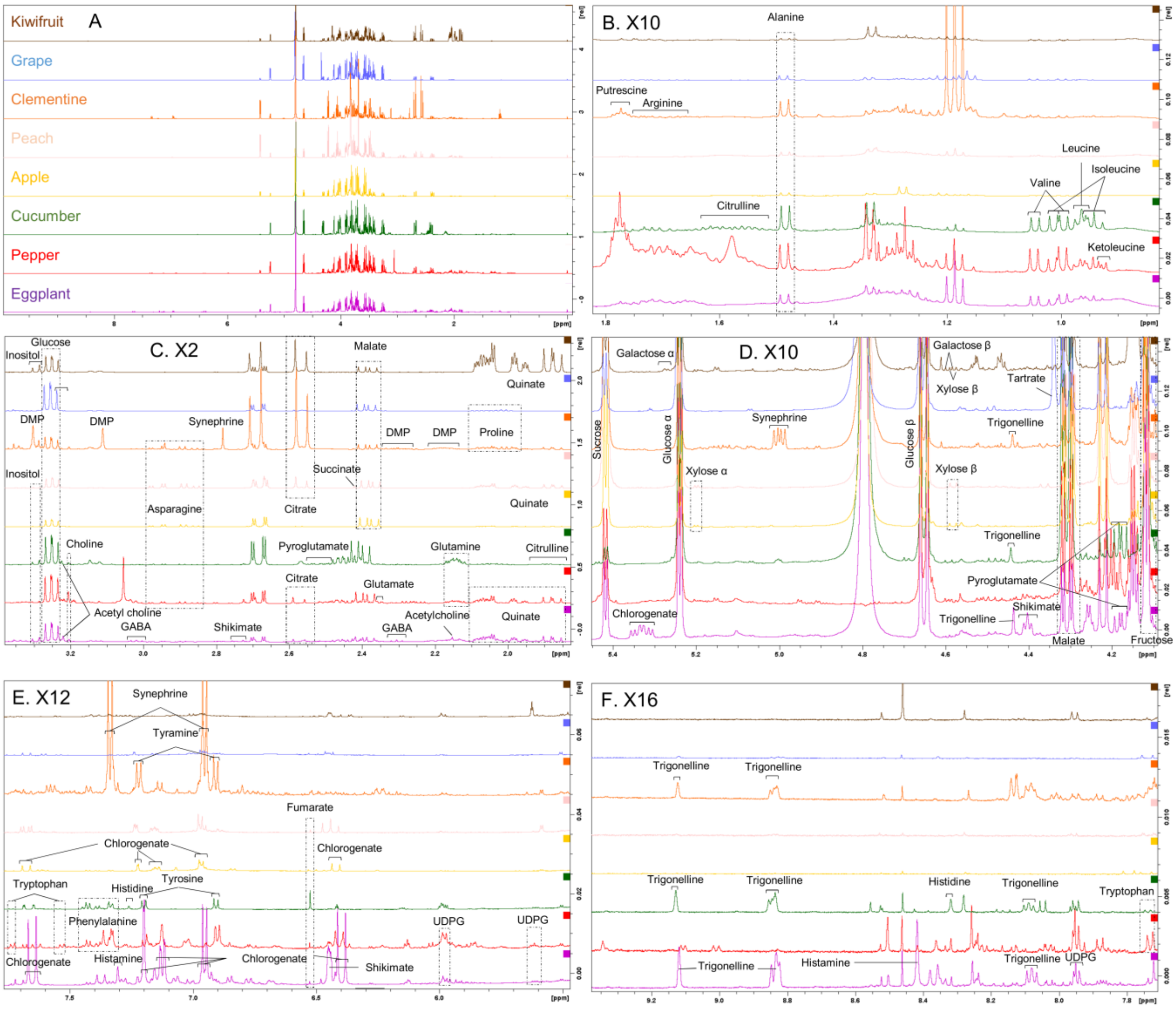

Figure S1. Annotated 1D ^{1}H-NMR spectra of polar extracts of the fruit mixed-stage samples. A. Overview of the eight spectra of the fruit species, (cpmg sequence was used for pepper extract and a zg sequence for other fruits). B. Zoom on spectra area between 0.85 and 1.8 ppm C. Zoom on spectra area between 1.85 and 3.35 ppm D. Zoom on spectra area between 4.1 and 5.4 ppm E. Zoom on spectra area between 5.5 and 7.7 ppm F. Zoom on spectra area between 7.7 and 9.35 ppm

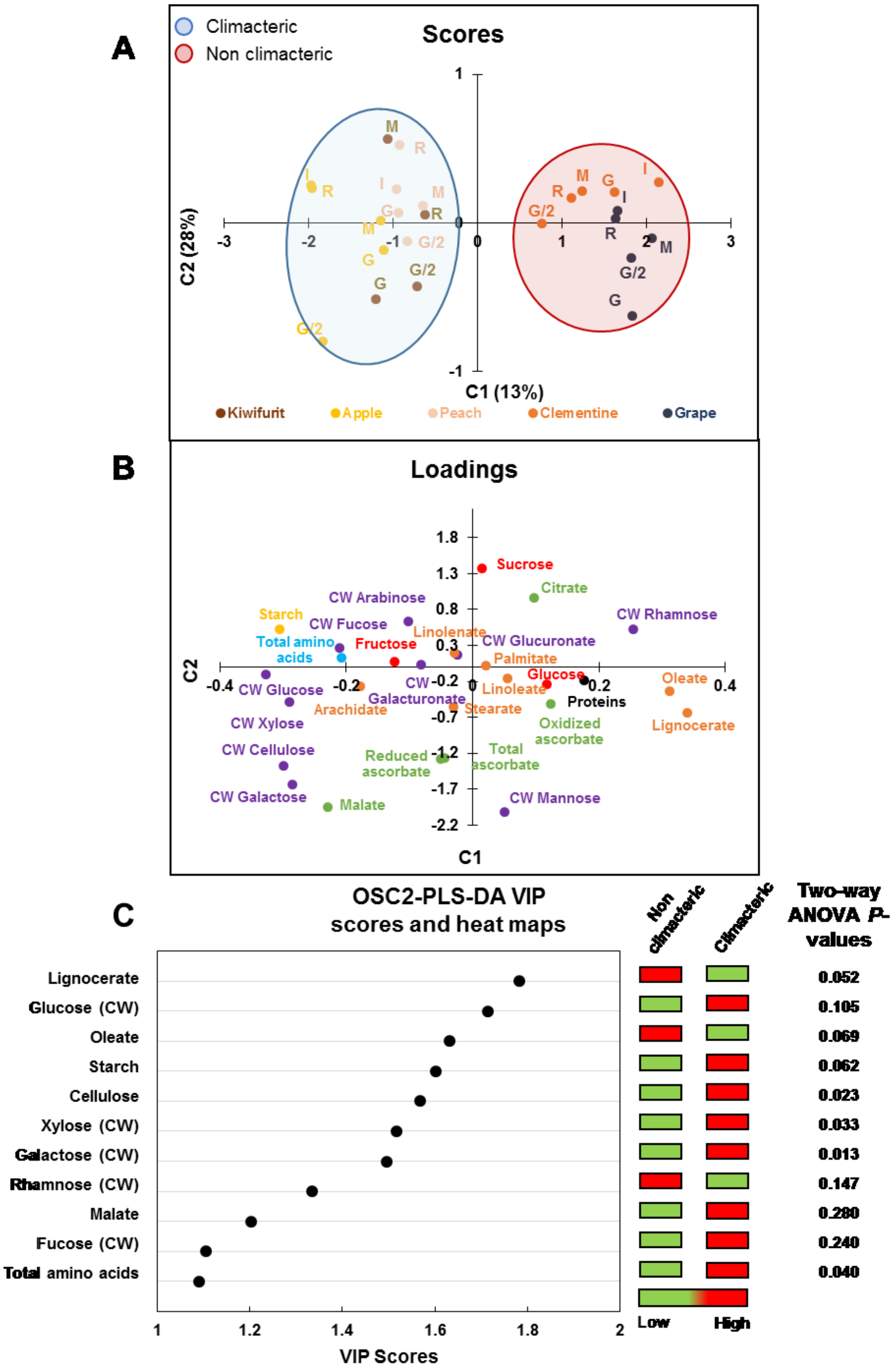

Figure S2. Discriminant analysis of three climacteric and two non-climacteric fruit species by OSC2-PLS-DA performed with 28 variables measured in 24 samples corresponding to four or five selected stages of development. A. Scores plot on the first two components. B. Loadings plot on the first two components. The compounds are coloured according to their biochemical family (red, soluble sugars; green, organic acids, blue, amino acids; orange, components of total lipids determined as FAMES; purple, components of cell-wall polysaccharides estimated by cell-wall monomers; yellow, starch; black total soluble proteins). Model quality parameters: $R^2Y = 90.6\%$, $Q^2 = 0.89$, P-value=0.04. C. Representation of VIP scores > 1 and associated heat maps of the averages of the 5 stages of development for 5 fruits with green color for low mean-values and red color for high mean-values. P-Values from two-way ANOVA.

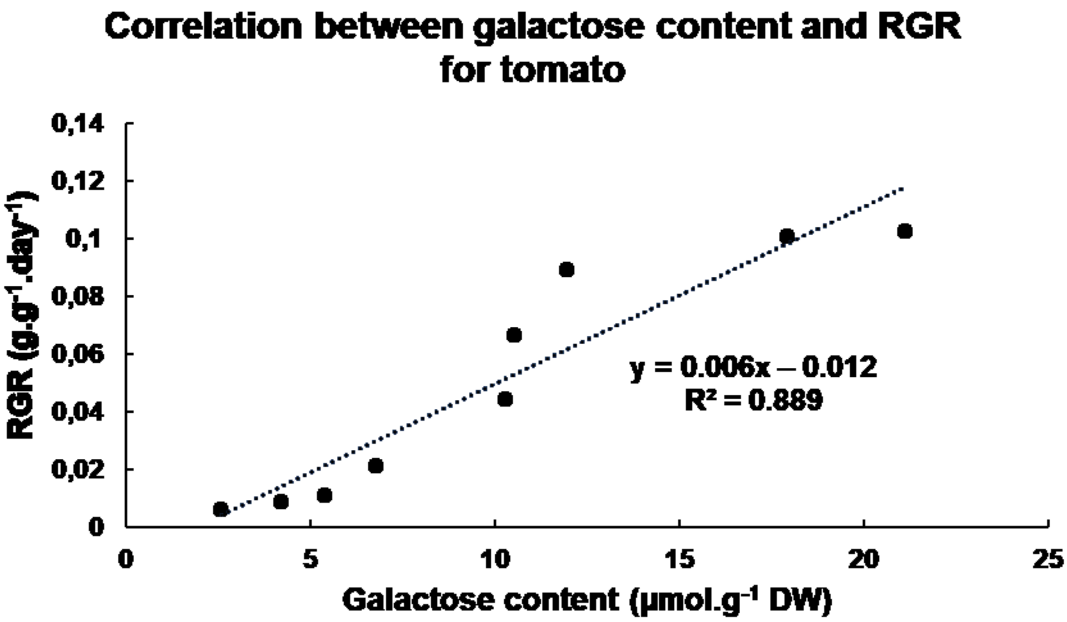

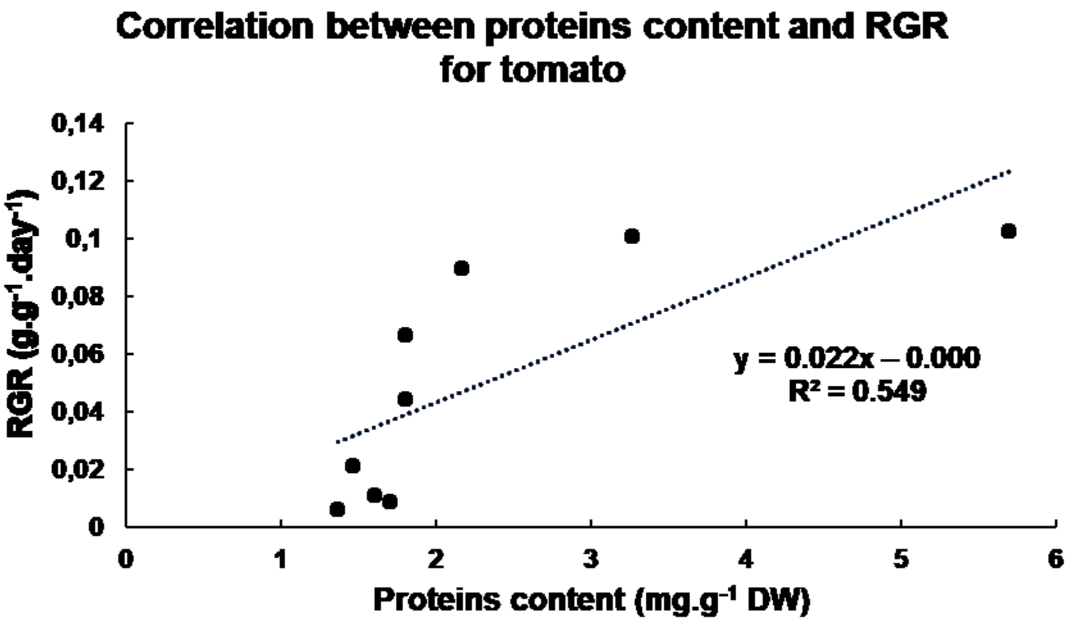

Figure S3. Correlation between cell-wall galactose or total soluble protein content and fruit RGR in tomato. Galactose content is expressed in µmol.g^{-1} DW, total soluble protein content in mg.g^{-1} DW and RGR in g.gFW^{-1}.day^{-1}. Total soluble protein content and fruit growth data are from Biais et al. (2014), and galactose from Colombié et al. (2015). RGR was recalculated from Biais et al. data using the approach used in the present work.
